# Towards the Elucidation of Assimilative *nasABC* Operon Transcriptional Regulation in *Haloferax mediterranei*

**DOI:** 10.3390/genes12050619

**Published:** 2021-04-22

**Authors:** Sandra Pastor-Soler, Mónica Camacho, Vanesa Bautista, María-José Bonete, Julia Esclapez

**Affiliations:** Agrochemistry and Biochemistry Department, Biochemistry and Molecular Biology Division, Faculty of Science, University of Alicante, Ap 99, E-03080 Alicante, Spain; sandra.pastor@ua.es (S.P.-S.); camacho@ua.es (M.C.); vanesa.bautista@ua.es (V.B.); julia.esclapez@ua.es (J.E.)

**Keywords:** haloarchaea, nitrogen assimilation, transcriptional regulation, DNA–protein pull-down

## Abstract

The assimilatory pathway of the nitrogen cycle in the haloarchaeon *Haloferax mediterranei* has been well described and characterized in previous studies. However, the regulatory mechanisms involved in the gene expression of this pathway remain unknown in haloarchaea. This work focuses on elucidating the regulation at the transcriptional level of the assimilative *nasABC* operon (HFX_2002 to HFX_2004) through different approaches. Characterization of its promoter region using β-galactosidase as a reporter gene and site-directed mutagenesis has allowed us to identify possible candidate binding regions for a transcriptional factor. The identification of a potential transcriptional regulator related to nitrogen metabolism has become a real challenge due to the lack of information on haloarchaea. The investigation of protein–DNA binding by streptavidin bead pull-down analysis combined with mass spectrometry resulted in the in vitro identification of a transcriptional regulator belonging to the Lrp/AsnC family, which binds to the *nasABC* operon promoter (p.*nasABC*). To our knowledge, this study is the first report to suggest the AsnC transcriptional regulator as a powerful candidate to play a regulatory role in *nasABC* gene expression in *Hfx. mediterranei* and, in general, in the assimilatory nitrogen pathway.

## 1. Introduction

The Archaea domain has basal transcription machinery similar in structure and organization to eukaryotes [1,2]. However, transcription regulation in Archaea is carried out by transcriptional factors (TFs) similar to Bacteria domain TFs. Indeed, 53% of archaeal TFs present a homolog in bacteria, in contrast to 2% of homologs identified in the Eukarya domain [3]. The most important archaeal regulators belong to the Lrp/AsnC, MarR, Ars/SmtB and TrmB families of transcriptional factors with an average of 9.6, 8.3, 4.6 and 4.3 members per genome, respectively [4]. Most of these factors consist of two domains: a DNA-binding domain and a ligand-binding domain. Like their bacterial homolog, the Archaea transcriptional regulators establish sequence-specific interactions between their DNA-binding domains and DNA-specific sequences which generally correspond to palindromic sequences 11–17 bp in size [5]. Moreover, the binding of transcriptional regulators to a single DNA site is exceptional because most of these regulators recognize several binding sites. Due to this multiple binding, affinity and sensitivity are increased, leading to a nonlinear regulatory response. Despite the recognition of several DNA-binding sites, there is often one central site in charge of regulation. In contrast, the other sites act as auxiliary operators that occupy the main regulatory site and contribute, to a lesser extent, to gene repression or activation processes [6]. Furthermore, there are also spatial limitations in the location of multiple binding sites dependent on DNA topology [7,8]. In this sense, several genes have been described in halophilic Archaea whose expression is affected by the level of DNA supercoiling and by alterations of structural transitions in the promoter regions [9,10,11,12,13], as well as the presence of histone-like proteins that allow differential packaging of DNA [14]. The nature of the transcriptional regulators also influences transcriptional regulation as they can act as repressors or activators depending on whether they decrease or increase mRNA levels, respectively [6]. Apart from gene regulation mediated by traditional transcriptional factors, the latest studies focus on identifying and analyzing the regulatory role of small RNAs (sRNAs). In Archaea, they are involved in many biological processes, including metabolic regulation, adaptation to extreme conditions, stress responses, morphology regulation, and cellular behavior [15,16,17].

This study focuses on the regulation at the transcriptional level of the nitrogen cycle’s assimilation pathway in *Hfx. mediterranei.* Particularly, we analyze the first stage of this pathway, which consists of the conversion of nitrate to nitrite via the assimilative nitrate reductase enzyme (Nas) encoded for the *nasA* gene [18]. The *nasA* gene (HFX_2002) is encoded in the *nasABC* operon where *nasB* (HFX_2003) codes for a membrane protein with similarity to the NarK transporter and *nasC* (HFX_2004) encodes a protein with similarity to MobA. Previous studies indicate that the *nasABC* genes are transcribed as one polycistronic messenger, while *nasD* (HFX_2005), which encodes an assimilative nitrite reductase, is transcribed as one monocistronic unit under the control of a different promoter region [19]. Regarding the transcriptional regulation of the *nasA* gene in *Hfx. mediterranei*, the presence of palindromic sequences in the *nasABC* promoter region [19] should be highlighted because they could serve as potential binding sites for transcriptional regulators that determine *nasABC* gene expression, as seen in other microorganisms belonging to the Archaea domain. The assimilation pathway of the nitrogen cycle is regulated according to the nitrogen source. Previous studies using RT-PCR, quantitative polymerase chain reaction (qPCR), and Northern blot allowed us to identify the absence of the transcription of *nasABC* and *nasD* in defined media with ammonium as a nitrogen source and the transcription of *nasABC* and *nasD* in defined media with nitrate or upon nitrogen starvation. Therefore, these results indicate this pathway is generally controlled according to the availability of ammonium [19,20,21]. The latest studies use microarrays for the analysis of the *Hfx. mediterranei* transcriptome according to the nitrogen source [22]. The results regarding the expression of the *nasABC* operon and the *nasD* gene support previous studies [19,20,21], indicating that the absence of ammonium is the triggering factor for the expression of genes of the nitrate assimilation pathway, which are overexpressed in the presence of nitrate or under conditions of nitrogen starvation. However, the molecular mechanisms responsible for the different gene expression patterns according to the nitrogen source remain unknown in Haloarchaea.

This work aims to be a starting point to understand regulation at the transcriptional level of the *Hfx. mediterranei nasABC* operon. We use two different approaches: the characterization and site-directed mutagenesis of the *nasABC* promoter region to identify potential binding sites of transcriptional factors and the optimization of DNA–protein pull-down assays to identify a transcriptional factor candidate involved in the regulation of nitrogen metabolism. As a result, a transcriptional factor belonging to the Lrp/AsnC family that recognizes and interacts with p.*nasABC* has been identified.

## 2. Materials and Methods

### 2.1. Strains and Growth Conditions

The bacterial strains used in this study featured *Escherichia coli* DH5α as a host of plasmid pGEM-T Easy (Promega, Madison, WI, USA), the dam-negative *E. coli* JM110 strain (to avoid the methylation of DNA), and *E. coli* XL2-Blue as a host strain deficient in all known restriction systems, preventing the cleavage of cloned DNA. These strains were grown aerobically in LB at 37 °C, and for the selection of positive transformants, ampicillin was added at a concentration of 100 µg·mL^−1^.

*Hfx. mediterranei* strain R4 (ATCC 33500^T^) was grown in defined culture media at pH 7.3 with 25% (*w*/*v*) seawater [23], adding 0.5 g/L KH_2_PO_4_, 5 g/L glucose, and 0.005 g/L FeCl_3_. Different nitrogen sources such as KNO_3_ and NH_4_Cl were used at a final concentration of 40 mM. Nitrogen starvation was facilitated by growing *Hfx. mediterranei* using ammonium as the nitrogen source with 0.6 OD_600nm_ (40 h of growth approximately); then, cells were harvested via centrifugation, washed with 20% of seawater, and transferred to a medium lacking a nitrogen source [22]. *Hfx. mediterranei* was grown at 42 °C in aerobic conditions at 180 rpm. Three independent biological replicates were performed for each condition to ensure reproducibility.

### 2.2. Constructs Used for Transformation

The promoter of *nasABC* genes (p.*nasABC*) was amplified via PCR using the oligonucleotides p.nas_for and p.nas_rev (p.nas_for, 5′-CAT CCC CCC AAG CTT GCC GGG GCA G-3′ and p.nas_rev, 5′-ACC ACA CCC CAT GGC ACA GCG CAT-3′), which included the appropriate target of the restriction enzymes *Nco*I and *Hind*III. This promoter was cloned in pGEM-T Easy Vector Systems (Promega, Madison, WI, USA) and subsequently into the pVA513 halophilic expression vector, which was kindly provided by Prof. Mike Dyall-Smith (of the University of Melbourne, Australia). The plasmid pVA513 ( bp) (Figure S1) contains the *E. coli* pBR322 plasmid *ori* region and ampicillin resistance (Amp^R^) gene, as well as the *Haloferax* pHK2 replicon region and novobiocin-resistance (Novo^R^) gene, enabling maintenance and selection in both hosts. Moreover, this vector has restriction sites for enzymes *Hind*III and *Nco*I to allow the cloning of the promoter region upstream of the *Haloferax lucentense bgaH* gene, which codifies β-galactosidase. Finally, the construction pVA-p.*nas* obtained was transformed in *Hfx. mediterranei* [24,25].

The different mutated versions of pVA-p.*nasABC* have been constructed using the oligonucleotides summarized in Table S1.

### 2.3. Site-Directed Mutagenesis

The different mutants of p.*nasABC* were produced as described previously [26] using *Pfu Turbo* DNA polymerase (Stratagene, La Jolla, CA, USA), which amplified different versions of the promoter region using mutated oligonucleotides (Table S1), and *Dpn*I (Fermentas, Thermo Fisher Scientific Inc., Madrid, Spain) for methylated parental vector digestion.

### 2.4. Transformation of Hfx. mediterranei and β–Galactosidase Assay

*Hfx. mediterranei* R4 was transformed with different versions of pVA-p.*nasABC* constructions as described previously [25]. Transformants selection was carried out using agar plates with novobiocin at a final concentration of 0.3 μg/mL, and positive transformants were analyzed via restriction analysis. β-galactosidase activity was determined by the o-nitrophenyl-β-d-galactopyranoside (ONPG) assay using 50 mM Tris-HCl pH 7.2, 2.5 M NaCl, 10 μM MnCl_2_, 0.1% β-mercaptoethanol as a *bgaH* activity buffer (bgaH buffer) [27]. The extract was incubated for 3 min at 40 °C with ONPG, and the absorbance changes at 405 nm were recorded for 5 min to measure β-galactosidase activity. BgaH buffer was used as a blank, and activity measurements were performed in triplicate in all cases. The protein concentrations of these extracts were determined using the Bradford method [28]. The results of β-galactosidase activity at different optical densities were represented in graphs using the GraphPad Prism 8 software.

### 2.5. DNA–Protein Interaction Analysis

The DNA–protein interaction assay was optimized for halophilic Archaea at different levels: the concentration of biotinylated DNA; the buffer used in DNA–magnetic bead binding; and the protein sample and elution buffer.

#### 2.5.1. Concentration of Biotinylated DNA Optimization

To test the protein binding affinity of the target DNA, p.*nasABC*, a pull-down assay was performed in parallel using three different DNA samples: (i) the p.*nasABC* promoter region (420 bp) as the target DNA; (ii) another halophilic promoter region (ferredoxin promoter, p.*fdx*) (210 bp) as the negative control; and (iii) fragment DNA (glucose dehydrogenase gene) (367 bp) as a second negative control. A fourth sample was tested as a third negative control in which the complete procedure was conducted in the absence of DNA. The biotinylated DNA for each condition was obtained via PCR using one of the oligonucleotides labeled with biotin (Table S1).

The maximum biotin-DNA concentration bound to the magnetic beads was optimized using a range of biotinylated DNA concentrations between 200 and 1200 pmol/mL in the presence of 20 mM Tris-HCl pH 7.5, 2 M NaCl, and 25 mM MgCl_2_ as a binding buffer. The results were checked using 1% agarose gel.

#### 2.5.2. Optimization of Pull-Down Assay Conditions: Protein Sample and Elution Buffer

Cell extracts at 60% (*w*/*v*) of *Hfx. mediterranei* R4, grown until the initial exponential phase in different defined media (with 40 mM nitrate or 40 mM ammonium as a nitrogen source, and in nitrogen starvation), were performed using different buffer compositions (Table 1).

The buffer composition used in the first elution was also optimized (Table 1). A second elution was performed using 1% SDS.

#### 2.5.3. Pull-Down Assay Adapted for Haloarchaea

For the binding of biotin-labeled DNA to streptavidin-charged magnetic beads, biotinylated DNA at a final concentration of 800 pmol/mL was incubated in the presence of the optimized binding buffer (20 mM Tris-HCl pH 7.5, 2 M NaCl, 25 mM MgCl_2_) with 50 μL of streptavidin-charged magnetic beads for 1 h with gentle shaking at room temperature. Next, the DNA-magnetic bead complex was incubated with the protein extract and shook for 3 h at 4 °C. After different tests, the buffer used to carry out the protein extract was 10 mM HEPES pH 7.5, 3 M KCl, 1 M NaCl, and 5 mM MgCl_2_. Subsequently, two serial elutions were performed, the first at 30 °C using 10 mM HEPES pH 7.5, 0.5 M KCl, 0.2 M NaCl, 5 mM MgCl_2_ buffer, and the second elution at 55 °C using 1% SDS solution. The samples of the different elutions were analyzed using SDS-PAGE with subsequent silver staining.

#### 2.5.4. Mass Spectrometry Analysis

After using SDS-PAGE, bands present only in the elutions of the assays performed with the DNA of interest, p*.nasABC*, were analyzed via mass spectrometry. The selected bands were digested with trypsin using the ProGest digestion kit (Genomic solutions, Cambridgeshire, UK) following the manufacturer’s recommendations. The samples were then washed with 25 mM ammonium bicarbonate to remove impurities and treated with 10 mM DTT and 100 mM iodoacetamide. The samples were digested with modified porcine trypsin (Promega, Madison, WI, USA) at 37 °C for 6–7 h. The digested peptides were extracted with ammonium bicarbonate to be subsequently treated with 70% acetonitrile and 1% formic acid. Once the sample was prepared, nano-ESI LC-MS/MS analysis was carried out via the Research Technical Services (SSTTI) from the University of Alicante and analyzed with Spectrum Mill software (Agilent, Santa Clara, CA, USA).

## 3. Results

### 3.1. Characterization of p.nasABC from Hfx. mediterranei

**Figure 1:**
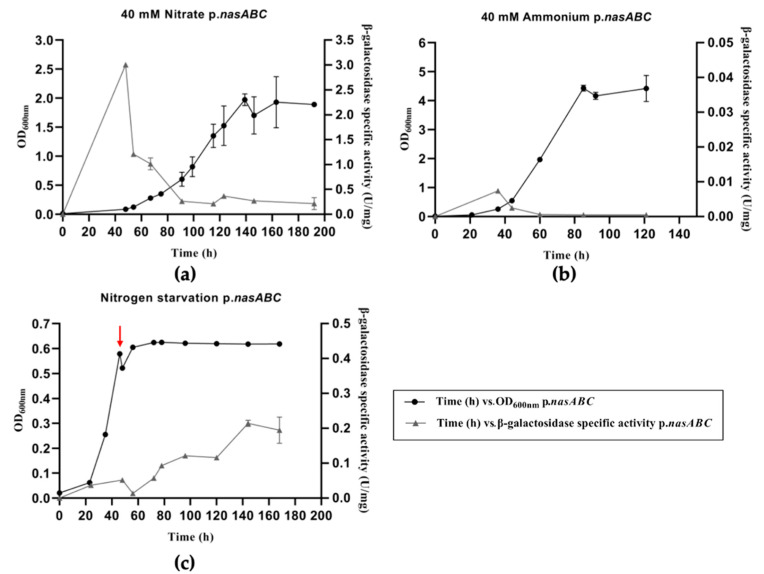
Cell growth, followed by measuring OD_600nm_, and β-galactosidase-specific activity, determined for *Hfx. mediterranei* p.*nasABC* transformants in two different defined media supplied with 40 mM nitrate (**a**) and 40 mM ammonium (**b**) as a nitrogen source. The third sample was kept under nitrogen-starved conditions (**c**). Timepoint where nitrogen starvation starts is indicated with a red arrow.

*nasABC* promoter characterization was carried out using the β-galactosidase (*bgaH*) gene from *Haloferax lucentense* as a reporter gene. Transformants of *Hfx. mediterranei* with pVA-p.*nasABC* were grown in defined culture media with 40 mM KNO_3_ or 40 mM NH_4_Cl as nitrogen sources and under nitrogen starvation conditions. The cultures were monitored at an optical density of 600 nm and following β-galactosidase-specific activity, as described previously (Figure 1).

The growth curves of *Hfx. mediterranei* pVA-p.*nasABC* transformants in defined media with nitrate or ammonium as a nitrogen source and under nitrogen starvation conditions (Figure 1) corresponded to those previously obtained [21]. The best growth of *Hfx. mediterranei* is obtained using ammonium as a nitrogen source along with other nitrogen compounds, such as nitrate.

When the transformant was grown in the presence of nitrate, the β-galactosidase activity presented a rapid increase at the lag phase and decreased at the beginning of the exponential growth phase, reaching its maximum value around 0.1 OD_600nm_. Then, the activity began to decrease during the exponential phase, showing residual activity in the stationary growth phase. However, the β-galactosidase activity profile obtained when *Hfx. mediterranei* pVA-p.*nasABC* transformants were grown in a defined media with ammonium as a nitrogen source (Figure 1b) was different. The β-galactosidase activity levels were close to zero throughout the time that the cultures were studied.

The effect of nitrogen starvation on *bgaH* activity is shown in Figure 1c. It was very low before inducing the nitrogen starvation; however, after 54 h, the activity increased, reaching a maximum activity of 0.2 U/mg at 140 h of starvation. Despite obtaining β-galactosidase activity under these culture conditions, its levels were approximately six times lower than those obtained in a defined medium with nitrate as a nitrogen source. The β-galactosidase-specific activity results revealed that the *nasABC* genes would be expressed under these limiting conditions, which are consistent with previous studies [21,22].

### 3.2. Mutagenesis and Characterization of the p.nasABC Using bgaH Activity

**Figure 2:**
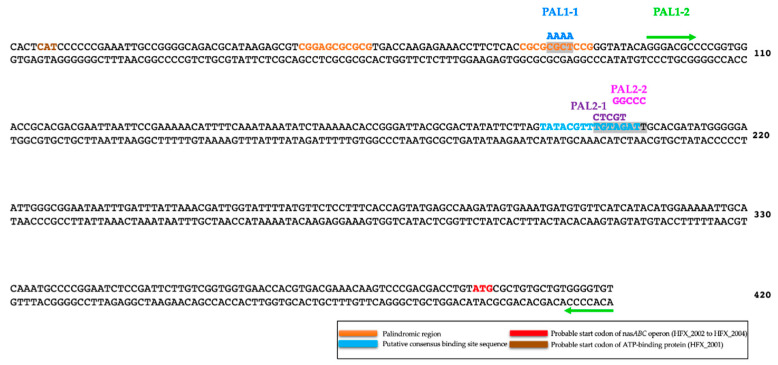
An illustration of the p.*nasABC* region from *Hfx. mediterranei*. Palindromic region 1 is highlighted in orange color, the putative consensus binding site sequence in light blue color, the probable start codon of *nasABC* operon is highlighted in red color and the probable start codon of a divergently encoded gene, which codifies an ATP-binding protein, is highlighted in brown color; the modifications introduced in the sequence are also shown.

As shown in Figure 2, the p.*nasABC* promoter presents two different regions, which after modification could be a promising target to help identify potential transcriptional regulator binding sites. These regions are a palindromic sequence (region 1) and a putative consensus sequence recognized by regulatory proteins (region 2). Four mutants of the p.*nasABC* sequence have been made: PAL1-1, PAL1-2, PAL2-1 and PAL2-2 (Figure 2). PAL1-1 and PAL1-2 present mutations in the palindromic sequence of the promoter (region 1). Five nucleotides have been modified in PAL1-1 to interrupt the palindrome, and in PAL1-2, the palindromic sequence has been completely left out. In PAL2-1 and PAL2-2, the putative consensus sequence of the promoter (region 2) has been modified, changing five nucleotides in each one. Therefore, none of the different p.*nasABC* mutants’ sequence modifications are expected to interfere with the transcription machinery.

The four mutants of p.*nasABC* were cloned into pVA513, creating the constructions pVA-PAL1-1 (PAL1-1), pVA-PAL1-2 (PAL1-2), pVA-PAL2-1(PAL2-1) and pVA-PAL2-2 (PAL2-2) (Figure S2). These constructions were successfully transformed in *Hfx. mediterranei* R4, except for pVA-PAL2-1.

#### 3.2.1. Analysis of Palindromic Region 1

**Figure 3:**
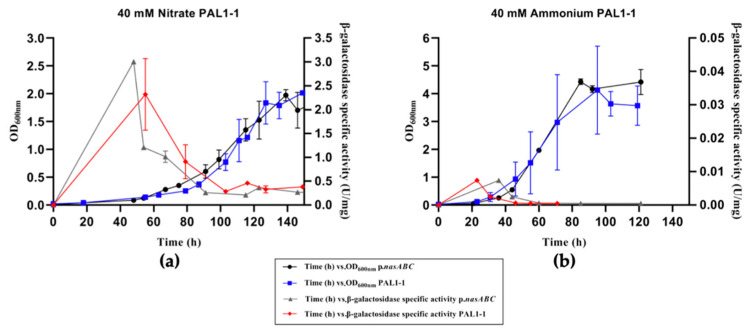
Cell growth, followed by measuring OD_600nm_, and β-galactosidase specific activity, determined for *Hfx. mediterranei* p.*nasABC* PAL1-1 transformants in defined media supplied with different nitrogen sources: 40 mM nitrate PAL1-1 (**a**) and 40 mM ammonium PAL1-1 (**b**).

**Figure 4:**
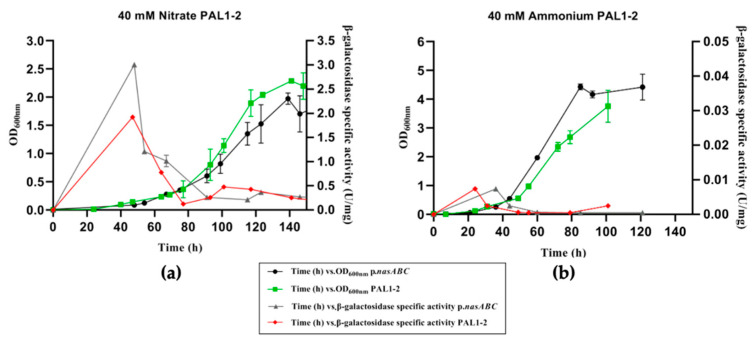
Cell growth, followed by measuring OD_600nm_, and β-galactosidase-specific activity, determined for *Hfx. mediterranei* p.*nasABC* PAL1-2 transformants in defined media supplied with different nitrogen sources: 40 mM nitrate PAL1-2 (**a**) and 40 mM ammonium PAL1-2 (**b**).

Two different modifications have been carried out in the palindromic region 1 as described above. *Hfx. mediterranei* transformants were analyzed following the methodology described previously. Figure 3 and Figure 4 show the growth of the transformants and their *bgaH*-specific activity in defined media with nitrate and ammonium as a nitrogen source.

In defined media with nitrate, PAL1-1 and PAL1-2 transformants showed the same growth and β-galactosidase-specific activity (U/mg) profile as p.*nasABC* transformants (Figure 3a and Figure 4a). Both followed a typical growth profile in the presence of nitrate as a nitrogen source, showing a more extended adaptation phase at the beginning of the growth period that was non-existent when using other nitrogen sources such as ammonium (Figure 3b and Figure 4b) [21].

The β-galactosidase-specific activity of the PAL1-1 and PAL1-2 transformants did not present significant trend differences compared to p.*nasABC*, suggesting that the sequence modifications carried out in palindromic sequence 1 did not affect the binding area of possible transcriptional regulators because the β-galactosidase-specific activity (U/mg) did not show significant variations. As in the case of p.*nasABC*, β-galactosidase activity experienced an abrupt and rapid increase during the first 50 h of growth, corresponding to the beginning of the exponential phase. The activity began to decrease during the end of the exponential phase and stayed low in the stationary phase.

In relation to the characterization of PAL1-1 and PAL1-2 in the defined medium with ammonium as the nitrogen source, the growth profiles and β-galactosidase activity did not show significant differences, with β-galactosidase activity not being detected as in the case of p.*nasABC*. Additionally, β-galactosidase activity was checked under nitrogen starvation culture conditions in both transformants, PAL1-1 and PAL1-2, and similar values to those measured with the p.*nasABC* were obtained (Figure S3). Based on these results, it appears that these modified regions in PAL1-1 and PAL1-2 transformants are not involved in the binding of any transcriptional regulator implicated in the regulation of nitrogen metabolism. It is possible that this palindromic region could be involved in the expression regulation of a divergently encoded gene, which codifies an ATP-binding protein (HFX_2001), on the opposite strand of the *nasABC* operon.

#### 3.2.2. Analysis of Putative Consensus Binding Site Sequence (Region 2)

**Figure 5:**
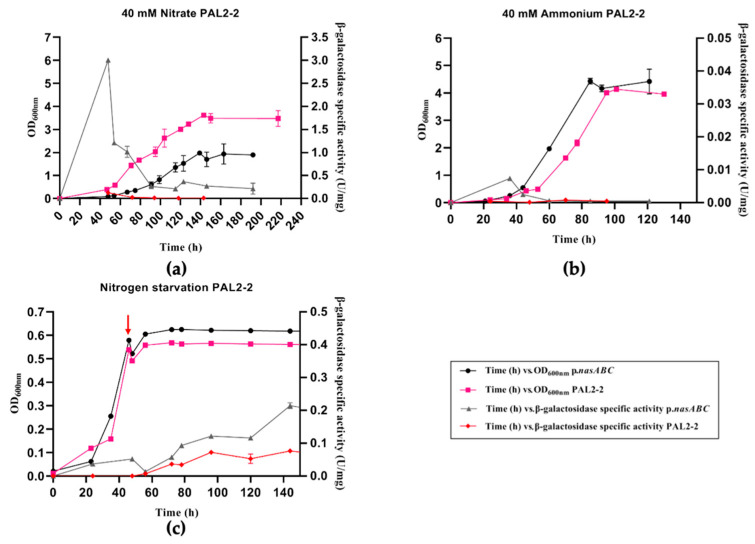
Cell growth, followed by measuring OD_600nm_, and β-galactosidase-specific activity, determined for the *Hfx. mediterranei* PAL2-2 transformant in two different defined media supplied with a nitrogen source of 40 mM nitrate PAL2-2 (**a**) and 40 mM ammonium PAL2-2 (**b**). The third sample was kept under nitrogen-starved conditions (**c**). Timepoint where nitrogen starvation starts is indicated with a red arrow.

Only the transformant PAL2-2 was successfully obtained, in which the putative consensus-binding site sequence was modified by the mutation of five nucleotides. This PAL2-2 transformant was characterized in a defined medium with ammonium and nitrate as the nitrogen sources, as well as under nitrogen starvation conditions (Figure 5).

PAL2-2 exhibited a typical growth trend in the presence of nitrate as a nitrogen source. The β-galactosidase activity in this transformant was practically undetectable (Figure 5a). This result indicated that the modification introduced in the putative consensus binding site sequence of the promoter affected a possible transcriptional regulator binding area, preventing its binding to the target promoter. Therefore, this sequence must play a key role in the regulation at the transcriptional level of the *nasABC* operon.

The characterization of the PAL2-2 transformant in defined media with ammonium as a nitrogen source did not show (Figure 5b) differences in the growth trend. PAL2-2 presented an exponential increase in growth until reaching a maximum growth at 90 h; from this point, it remained stable in the stationary phase with optical densities around 4. The PAL2-2 transformant β-galactosidase activity in the defined media with ammonium followed the same trend as p.*nasABC*. As in the other transformants (PAL1-1 and PAL1-2) (Figure 3a and Figure 4a), no significant differences in β-galactosidase activity were obtained concerning p.*nasABC*, presenting values close to zero throughout the growth of PAL2-2. These data are in agreement with previous works, where *nasABC* expression is repressed when ammonium is used as a nitrogen source [18,19,20,21,22].

In nitrogen starvation culture conditions, the PAL2-2 transformant (Figure 5c) shows a similar growth curve to p.*nasABC*. However, relevant differences have been observed in β-galactosidase activity under this condition. Its maximum activity is under 0.1 U/mg, reduced by more than 50% compared to the activity determined in pVA-p.*nasABC* transformants under the same condition.

### 3.3. Pull-Down Assay

Before carrying out the DNA–protein binding assay, optimization was needed. The maximum biotinylated DNA concentration capable of interacting with the magnetic beads was optimized, testing biotinylated DNA concentrations between 200 and 1200 pmol/mL and obtaining the best binding at 800 pmol/mL. Additionally, eight different buffers were tested for the *Hfx. mediterranei* protein extract preparation combined with five elution buffers, only obtaining successful results in one combination of these: 10 mM HEPES pH 7.5, 3 M KCl, 1 M NaCl, and 5 mM MgCl_2_ as the protein extract buffer and 10 mM HEPES pH 7.5, 0.5 M KCl, 0.2 M NaCl, and 5 mM MgCl_2_ as the elution buffer.

A DNA protein-binding assay was performed with *Hfx. mediterranei* R4 protein extracts from three cultures using different nitrogen sources: 40 mM ammonium, 40 mM nitrate, and nitrogen starvation. Even though different trials were conducted, no relevant results were obtained using the protein extracts from the culture grown under conditions of nitrogen starvation.

**Figure 6:**
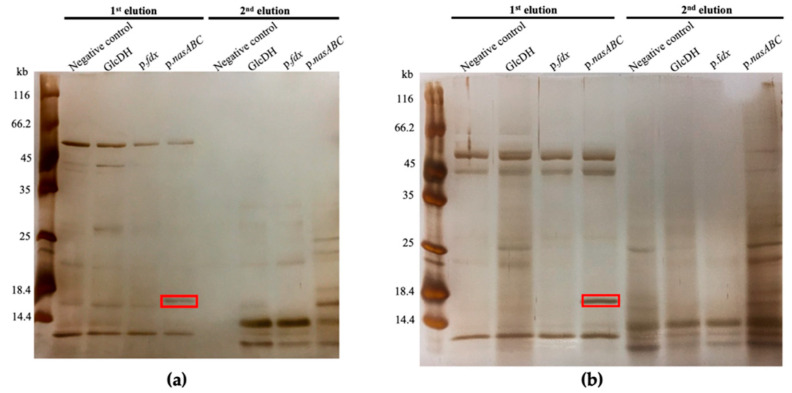
Silver staining of the pull-down assay with protein extracts of *Hfx. mediterranei* R4 in defined media with 40 mM nitrate (**a**) and 40 mM ammonium (**b**) using different fragments of biotinylated DNA (negative control; GlcDH; p.*fdx*; p.*nasABC*). Bands selected for analysis by mass spectrometry are highlighted in red.

Once the DNA–protein pull-down was performed with the different DNA samples (glucose dehydrogenase gene; p*.fdx*; p.*nasABC*), the elution fractions obtained were analyzed via SDS-PAGE and silver staining (Figure 6). Comparison of the protein band profiles in each line (controls and sample) allowed for the identification of the band, which only appears in the sample with the target DNA (p.*nasABC*). The negative control without DNA allowed us to identify and discard the possible unspecific protein binding to the DNA magnetic beads complex. The use of the glucose dehydrogenase biotinylated fragment allowed for verification of the unspecific union of a protein to any DNA fragment. The ferredoxin biotinylated promoter was used to test the unspecific recognition of the promoter sequences.

The SDS-PAGE analyses were performed with samples obtained from cultures grown in nitrate (Figure 6a) and ammonium (Figure 6b) as a nitrogen source. In both SDS-PAGE analyses, different bands of unspecific bound proteins were observed in the three control samples (the negative control, glucose dehydrogenase, and ferredoxin promoter). For identifying the bands susceptible to analysis via mass spectrometry (Agilent 1100 Series LC/MSD Trap SL), we searched the bands that were only present in the lines corresponding to the pull-down elutions using the biotinylated p.*nasABC* as a DNA sample. Under both growing conditions, a band of approximately 18 kDa was identified in the first elution of the pull-down assay using biotinylated p.*nasABC* (highlighted in red) and it was analyzed by mass spectrometry (Agilent 1100 Series LC/MSD Trap SL). The mass spectrometry results (Table 2) showed that this band corresponds to an Lrp/AsnC family transcriptional regulator (HFX_0246).

Therefore, these results indicated that an Lrp/AsnC transcriptional regulator family could interact in vitro with the *nasABC* promoter region and regulate the expression of *nasABC* genes in *Hfx. mediterranei*.

## 4. Discussion

Transcriptional regulators that control the expression of the nitrate assimilative pathway genes in bacteria have been highly studied; however, there is scarce information on the Archaea domain, and the regulation of nitrate assimilation genes has only been described in a few microorganisms [31]. The molecular mechanism of the regulation of this pathway has not been elucidated in *Hfx. mediterranei*, and no similar transcriptional regulator described for this pathway has been identified in its genome.

Characterization of the p*.nasABC* promoter using the *bgaH* gene of *Hfx. lucentense* as a reporter gene showed significant differences with the protein extracts from defined media with nitrate and under nitrogen-starved conditions. This allows us to presume the expression of the *nasABC* operon under these conditions and, consequently, the activation of the nitrogen cycle’s assimilative pathway. In contrast, no significant β-galactosidase activity was measured in cell extracts from cultures grown in a defined medium with ammonium as a nitrogen source. This suggests that in cultures with ammonium as a nitrogen source, the *nasABC* operon shows a low basal expression. These results supported the previous studies of RT-PCR and qPCR, where the transcripts of genes involved in the nitrogen assimilation pathway, like assimilative nitrate reductase, were expressed in media with nitrate as the nitrogen source [19,20]. Other results that support those obtained were the previous dot blot and Northern blot analyses, in which the exponential growth phase was related to *nasA* gene expression [21] as well as its enzyme activity [18]. Additionally, microarray studies showed an increased expression of the nitrogen assimilation pathway’s genes in the presence of nitrate as an inorganic nitrogen source or under nitrogen starvation conditions [22]. All these previous data are in good agreement with the characterization of p.*nasABC* using *bgaH* as a reporter gene, confirming that *nasABC* expression occurs in the presence of nitrate at the beginning of the exponential growth phase and nitrogen starvation because the microorganism needs to start its nitrate assimilation pathway. Therefore, high p.*nasABC* operon expression was obtained in the initial growth phase, which allows *Hfx. mediterranei* to metabolize the inorganic nitrogen source (nitrate) as quickly as possible to finally yield ammonium that will be made metabolically competent by GS and GOGAT in a process known as the GS/GOGAT cycle [32].

The characterization of the modified promoter region has allowed the identification of a potential DNA binding site for a possible regulatory protein involved in regulating the nitrate reductase operon. Significant results were only obtained when the sequence of region 2 was mutated (p.*nasABC*: 5′-TAGAT-3′; PAL2-2 transformant: 5′-GGCCC-3′), obtaining a marked decrease in β-galactosidase activity in defined media with nitrate as a nitrogen source. Therefore, a possible transcriptional regulator would bind in these conditions to this putative consensus-binding site sequence region and act as a transcriptional activator. This regulator would activate *nasABC* transcription when *Hfx. mediterranei* grows under nitrogen-limited conditions. In contrast, under nitrogen starvation culture conditions, PAL2-2 β-galactosidase activity was detected, but this was lower than in pVA-p.*nasABC*. Therefore, it could be the case that several transcriptional regulators or different regulation strategies are involved in the expression of the *nasABC* operon [22]. However, the β-galactosidase activity results obtained in the PAL2-2 transformant in the defined media with ammonium were similar to those obtained in p.*nasABC*, indicating that under these culture conditions, the expression of the *nasABC* gene cluster could be regulated by means of other strategies—for example, the binding of effector molecules.

The next step to study the regulation of the nitrate reductase operon in *Hfx. mediterranei* was to identify transcriptional regulators that would interact with the p.*nasABC* through a DNA protein pull-down assay, optimized in this study. This pull-down assay has been used for the first time to identify transcriptional regulators among all the existing proteins in the protein extract used in haloarchaea. It determined that the transcriptional regulator interacting with p.*nasABC* was a protein of the Lrp/AsnC transcriptional regulator family (HFX_0246). Strikingly, this Lrp/AsnC transcriptional regulator is encoded next to glutamine synthetase (GS), an enzyme involved in the nitrate assimilation pathway’s last steps. Previous studies in the Archaea *Halobacterium salinarum* have related this regulator’s function with GS expression [29]. Curiously, the ideal binding site of the Lrp transcriptional factor (5′-AGAATTTATTCT-3′) [33] is similar to the mutation in region 2 of the *nasABC* promoter, which results in decreased β-galactosidase activity in the presence of nitrate as the nitrogen source. The DNA-binding sequence specificities of Lrp regulators can vary widely and it is common that these sequences are quite degenerated [34]. In addition, not all the DNA binding sites must be near the TATA box; EMSA studies performed with Lrp from *Sulfolobus acidocaldarius* have revealed that the DNA binding site recognized by this transcriptional factor can be located away from the TATA box (188–212 nucleotides upstream of the translation start site) [35]. Consequently, it is possible that the Lrp/AsnC regulator identified via the pull-down assay interacts with the putative consensus-binding site sequence observed in the *nasABC* promoter region, as well as being involved in the modulation of this operon’s activity and, thus, in the regulation of the nitrogen assimilation pathway, as occurs in *Hbt. salinarum*.

This study demonstrated that the identified Lrp/AsnC family protein was expressed in nitrate and ammonium as a nitrogen source in vivo. Its expression under different culture conditions was expected because this family of transcriptional regulators regulates multiple physiological processes in other Archaea domain microorganisms, acting as global regulators of different biological processes [8,29,33,36,37,38,39,40]. In vitro, the Lrp/AsnC transcriptional regulator can establish an interaction with a region of the p.*nasABC*. However, the in vivo regulatory mechanism still remains unknown. Different effector molecules of the Lrp regulator induce conformational changes, which affect DNA binding and regulatory properties of the regulator according to environmental changes. A possible hypothesis to explain the results presented in this paper could be that, although Lrp/AsnC is expressed in the presence of nitrate and ammonium, effector binding could induce conformational changes which lead to either a decrease or increase in DNA-binding affinity and, subsequently, modulate the expression of the *nasABC* operon depending on nitrogen availability.

## 5. Conclusions

To conclude, a possible regulatory binding site has been identified by site-directed mutagenesis of the *nasABC* promoter region. Based on the investigation of β-galactosidase activity, the potential regulator could act as an activator. Furthermore, this is the first in vitro identification of a transcriptional regulator involved in nitrogen metabolism using crude extracts of a halophilic microorganism by a DNA–protein interaction assay. The optimization of this methodology has allowed the identification of a protein belonging to the Lrp/AsnC family as a potential transcriptional factor involved in regulating *nasABC* expression. Although more work is needed to elucidate the molecular mechanisms of *nasABC* expression, this work entails an excellent starting point to extend knowledge of the nitrogen regulation metabolism in Haloarchaea.

## Figures and Tables

**Table 1:** The buffers used for protein extraction and the first elution in pull-down assays *.

Protein Extract Buffer	1st Elution Buffer
20 mM Tris-HCl pH 7.5, 1 M NaCl, 25 mM MgCl_2_	20 mM Tris-HCl pH 7.5, 0.2 M NaCl, 25 mM MgCl_2_
20 mM Tris-HCl pH 7.5, 2 M NaCl, 25 mM MgCl_2_	
10 mM HEPES pH 7.5, 2 M KCl, 0.5 M NaCl, 5 mM MgCl_2_	**10 mM HEPES pH 7.5, 0.5 M KCl, 0.2 M NaCl, 5 mM MgCl_2_**
**10 mM HEPES pH 7.5, 3 M KCl, 1 M NaCl, 5 mM MgCl_2_** [29]	
20 mM Tris-HCl pH 7.5, 1 M KCl, 25 mM MgCl_2_	20 mM Tris-HCl pH 7.5, 0.2 M KCl, 25 mM MgCl_2_
20 mM Tris-HCl pH 7.5, 2 M KCl, 25 mM MgCl_2_	
10 mM Tris-HCl pH 7.5, 1 M NaCl	1% SDS
20 mM Tris-HCl pH 7.5, 10 mM EDTA, 80 mM (NH_4_)_2_SO_4_, 15% glycerol [30]	20 mM Tris-HCl pH 7.5, 10 mM EDTA, 380 mM NaCl, 15% glycerol

* Buffers printed in bold were found to be optimal.

**Table 2:** Mass spectrometry analysis of pull-down samples.

Culture Condition	Spectra	Distinct Peptides	Distinct Summed MS/MS Search Score	%AA Coverage	Total Protein Spectral Intensity	Species	NCBI Database	Protein Name
Nitrate 40 mM	9	3	48.81	22.3	4.69 × 10^8^	*Hfx. mediterranei* ATCC 33500	HFX_0246	Lrp/AsnC family transcriptional regulator
Ammonium 40 mM	22	5	75.89	40.7	4.67 × 10^8^	*Hfx. mediterranei* ATCC 33500	HFX_0246	Lrp/AsnC family transcriptional regulator

## Data Availability

The data presented in this study are available within the article.

## Supplementary Materials

The following are available online at https://www.mdpi.com/article/10.3390/genes12050619/s1, Table S1: Oligonucleotides designed for sited directed mutagenesis of p.*nasABC* region and for obtaining biotinylated DNA samples, Figure S1: pVA513 halophilic expression vector, which was kindly provided by Mike Dyall-Smith (University of Melbourne, Australia), Figure S2: Sequence of promoter region in pVA-PAL1-1 (a), pVA-PAL1-2 (b), pVA-PAL2-1(c), and pVA-PAL2-2 (d), Figure S3: Cell growth, followed by measuring OD_600nm_, and β-galactosidase specific activity, determined for *Hfx. mediterranei* p.*nasABC* PAL1-1 transformants (a) and PAL1-2 trans-formants (b) under nitrogen-starved conditions. Time point where nitrogen starvation starts is indicated with a red arrow.
