# Supplementary material for: Towards the Elucidation of Assimilative nasABC Operon Transcriptional Regulation in Haloferax mediterranei
Source: Genes (Basel). 2021 Apr 22;12(5):619. doi: 10.3390/genes12050619 (PMC8143581; doi:10.3390/genes12050619)
Supplement: Supplementary file 1 [file genes-12-00619-s001.zip › genes-1128334-suppl.pdf]

genes

MDPI

Table S1. Oligonucleotides designed for sited directed mutagenesis of p.*nasABC* region and for obtaining biotinylated DNA samples.

Oligonucleotide	Sequence	Target
PAL1-1_for	GAGAAACCTTCTCACCGCGAAAACCGGGTATACAGGGACGC	Modification of palindromic sequence by sited directed mutagenesis
PAL1-1_rev	GCGTCCCTGTATACCCGGTTTTCGCGGTGAGAAGGTTTCTC	
PAL1-2_for<br>p.nas_rev	GGGACGCCCCGGTGGACCGCACGACG<br>ACCACACCCCATGGCACAGCGCAT	Promoter amplification without palindromic region
PAL2-1_for	ATATTCTTAGTATACGTTCTCGTATTGCACGATATGGGGG	Modification of putative consensus binding site sequence by sited directed mutagenesis
PAL2-1_rev	CCCCCATATCGTGCAATACGAGAACGTATACTAAGAATAT	
PAL2-2_for	GTATACGTTTGTGGCCCGCACGATATGGGGGAATTGGGCGG	Modification of putative consensus binding site sequence by sited directed mutagenesis
PAL2-2_rev	CCGCCCAATTCCCCCATATCGTGCGGGCCACAAACGTATAC	
pVA-for-Biot<br>N-bGal-rev	GCTCGGAATTAACCCTCACTAAAG<br>GCTCCGGGAAATAGCAGACAC	Biotinylated p.*nasABC*
p.fdx_for<br>p.fdx_rev-Biot	TACTTCGTACGCGCTGTTCT<br>GTTACCGTGGGCATCAACAA	Biotinylated p.*fdx*
Glc_for<br>Glc_rev-Biot	GCATTGGTAAACATATGAAAGC<br>GTGAAGAACTGGACATGTAC	Biotinylated glucose dehydrogenase

The mutant sequences are shown in red color.

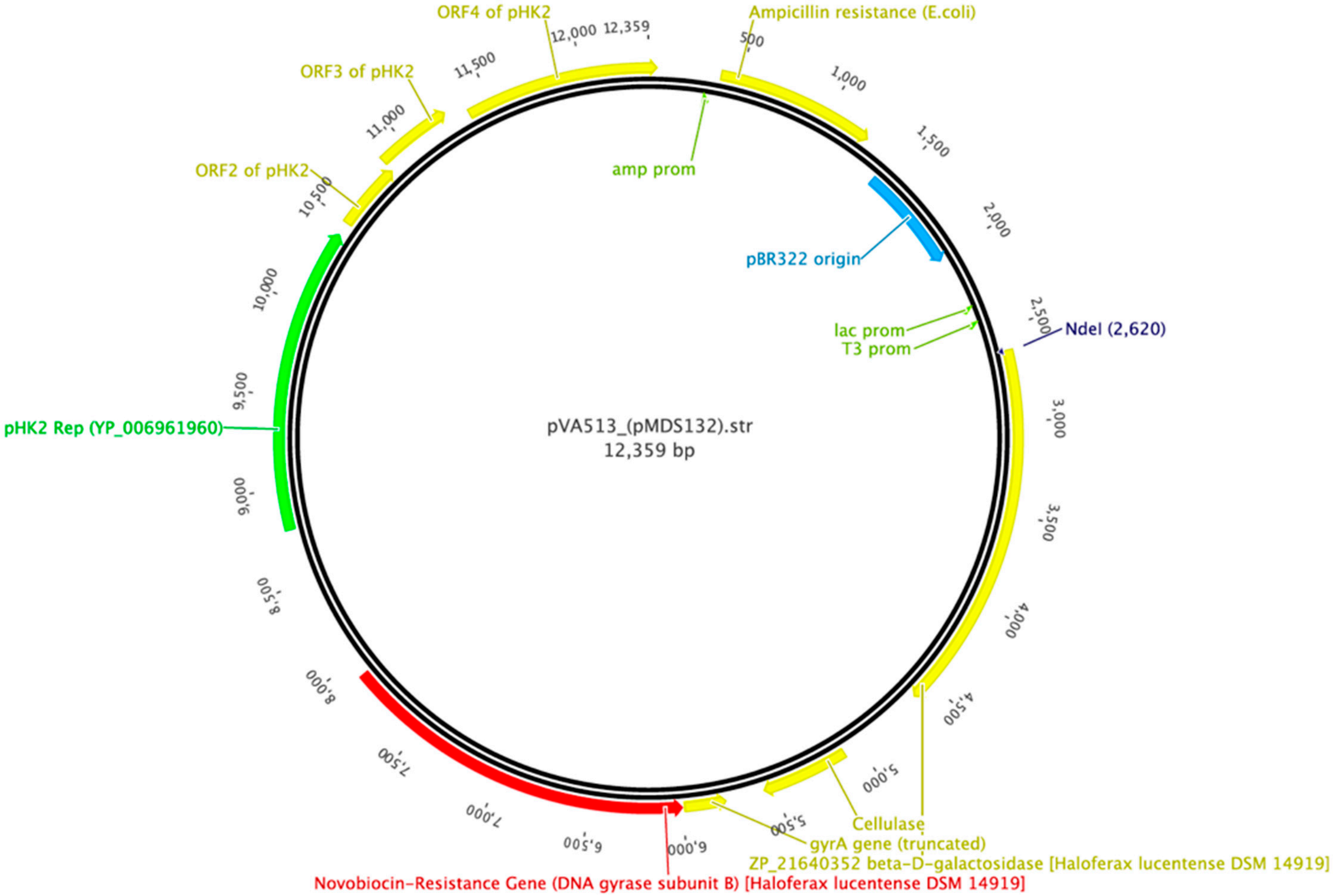

Figure S1. pVA513 halophilic expression vector, which was kindly provided by Mike Dyall-Smith (University of Melbourne, Australia).

Reference

Serrano-Gomicia, J.A. Ciclo del glioxilato en el arquea halófilo *Haloferax volcanii*: análisis bioquímico, filogenético y transcripcional. Ph.D. Dissertation, University of Alicante, Spain. 2000.

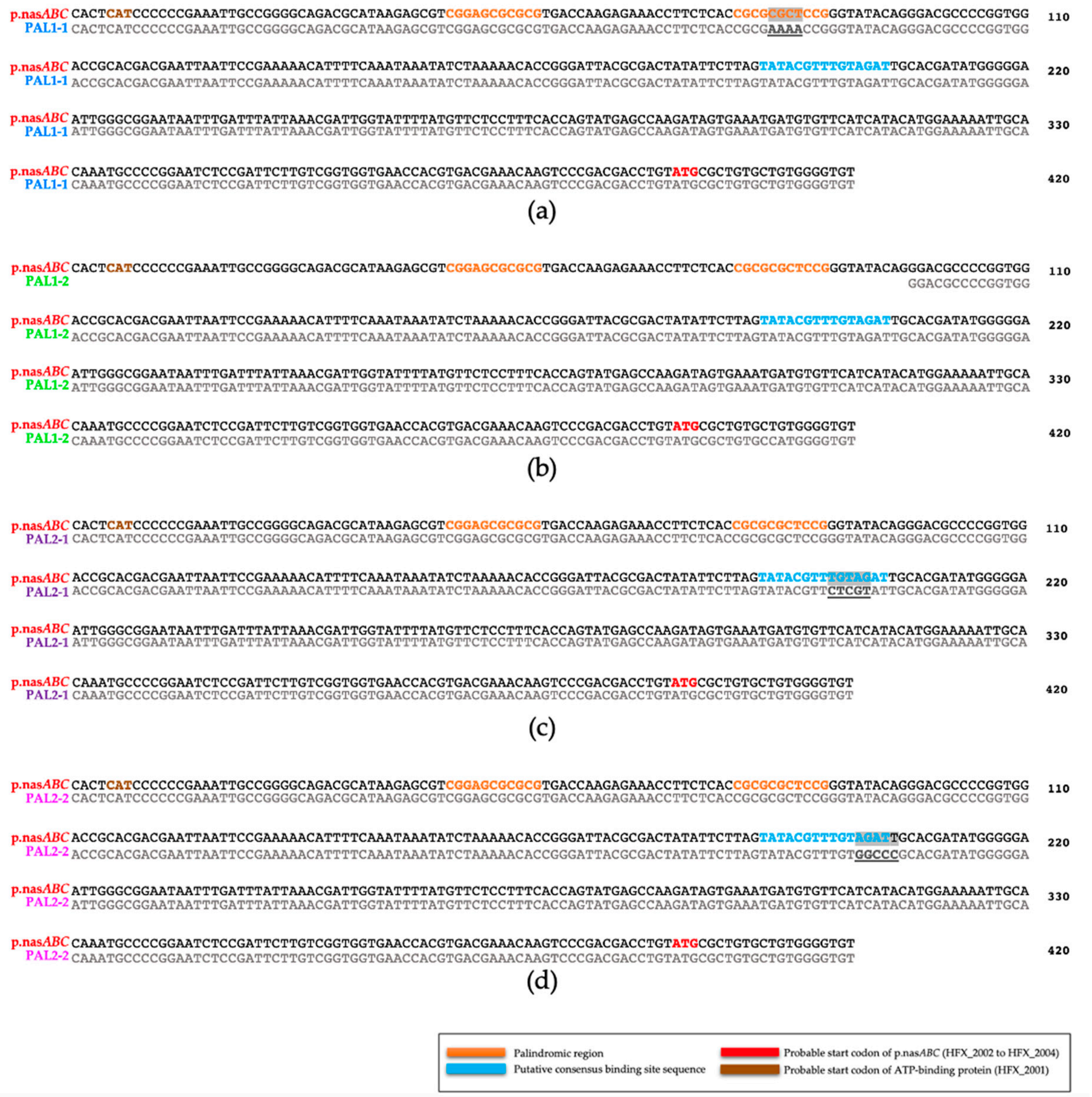

Figure S2. Sequence of promoter region in pVA-PAL1-1 (a), pVA-PAL1-2 (b), pVA-PAL2-1(c), and pVA-PAL2-2 (d).

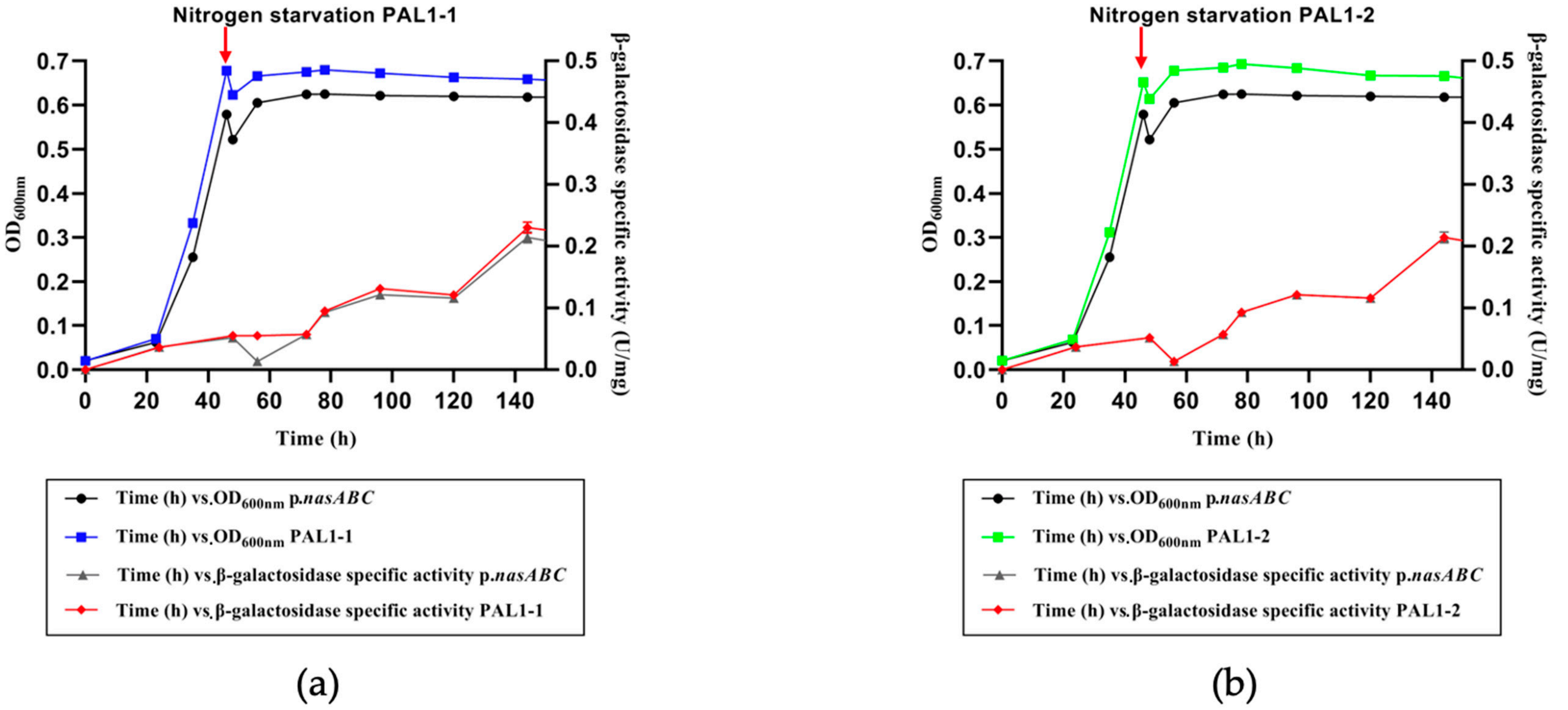

Figure S3. Cell growth, followed by measuring OD_{600nm}, and β-galactosidase specific activity, determined for *Hfx. mediterranei* p.*nasABC* PAL1-1 transformants (a) and PAL1-2 transformants (b) under nitrogen-starved conditions. Time point where nitrogen starvation starts is indicated with a red arrow.
